# Does precautionary information about electromagnetic fields trigger nocebo responses? An experimental risk communication study

**DOI:** 10.1186/s12940-018-0377-y

**Published:** 2018-04-12

**Authors:** Christoph Boehmert, Adam Verrender, Mario Pauli, Peter Wiedemann

**Affiliations:** 1 Department of Science Communication, Faculty for Humanities and Social Sciences, Karlsruhe Institute of Technology, Englerstraße 2, 76131 Karlsruhe, Germany; 2 Australian Centre for Electromagnetic Bioeffects Research, Wollongong, Australia; 3 School of Psychology, Illawarra Health & Medical Research Institute, University of Wollongong, Northfields Ave, Wollongong, NSW 2522 Australia; 4 Institute of Radio Frequency Engineering and Electronics (IHE), Karlsruhe Institute of Technology, Engesserstraße 5, 76131 Karlsruhe, Germany; 5 School of Psychology, Faculty of Social Sciences, University of Wollongong, Northfields Ave, Wollongong, NSW 2522 Australia

**Keywords:** Precaution, Nocebo effect, RF EMF, Risk communication, Risk perception

## Abstract

**Background:**

Regarding electromagnetic fields from mobile communication technologies, empirical studies have shown that precautionary information given to lay recipients increases their risk perceptions, i.e. the belief that electromagnetic fields are dangerous. Taking this finding one step further, the current study investigates whether precautionary information also leads to higher symptom perceptions in an alleged exposure situation. Building on existing research on nocebo responses to sham electromagnetic fields, an interaction of the precautionary information with personality characteristics was hypothesised.

**Methods:**

An experimental design with sham exposure to an electromagnetic field of a WLAN device was deployed. The final sample is constituted by *N* = 137 participants. Participants received either only basic information about the safety of current WLAN exposure limits or in addition also precautionary information (e.g. ‘prefer wired connections if wireless technology can be relinquished’). Subsequently, symptoms and other variables were assessed before and after sham exposure to a WLAN electromagnetic field.

**Results:**

Results are not in favour of the hypothesised effects. There was neither a main effect of precautionary information, nor were there any of the hypothesised interaction effects of precautionary information and personality characteristics on perceived symptoms under sham exposure. Exploratory analyses highlight the role of prior risk perception as a predictor of nocebo responses, and of symptom expectations as a mediator between these two variables.

**Conclusions:**

As the statistical power to detect even small effects was relatively high, we interpret this as a robust indication that precautionary information does not lead to increased nocebo responses by itself. The implications for health authorities´ communication with the public are discussed.

## Background

In many countries across the world, the precautionary principle is a cornerstone of radiation protection. This is especially true for non-ionizing radiation protection, i.e., regarding radio-frequency electromagnetic fields (RF EMFs) emitted by base stations, mobile phones and other wireless gadgets. The International Commission on Non-Ionising Radiation Protection (ICNIRP) emphasises that despite a substantial body of research, there is no conclusive evidence for any health effects of radiofrequency electromagnetic fields within the recommended exposure limits [1], a stance that has also been adopted by the World Health Organisation (WHO). However, the International Agency for Research on Cancer (IARC) has classified RF EMFS of mobile phones as a 2B “possible carcinogen” to humans, but emphasises that the evidence for an increase in glioma and acoustic neuroma among users of mobile phones was limited and that the evidence for an increase in other cancers was inadequate [2]. Most countries have adopted the exposure limits recommended by ICNIRP. In the face of the two differing assessments, RF EMF precautionary actions are recommended by many regulatory agencies and scientific organisations across the world (e.g. ARPANSA in Australia, ANSES in France, the German BfS, UK National Radiological Protection Board, now the UK Health Protection Agency, and the BAG in Switzerland). Usually, these approaches entail the recommendation of individual precautions. For instance, regarding Wireless Local Area Networks (WLAN), the German radiation protection agency (BfS) recommends to reduce exposure by using a LAN cable and by not installing WLAN-routers in places where people stay permanently ([3]; a translation can be found in Table 1). In some countries, further precautions are taken. For instance, in Switzerland stricter exposure limits have been set for mobile phone base stations and other stationary EMF-emitting antennas at so called places of sensitive use (for example apartments, schools, children’s playgrounds).

**Table 1:** Information about WLAN health effects and precautions used for experimental manipulation

Basic information	Precautionary information
**Are there health risks?** The specific absorption rate (SAR) is the basis for evaluating if high-frequency electromagnetic fields pose a health risk due to immediate effects. The SAR describes how much radiated power is absorbed by human body tissue in a given situation. For health protection, recommended limit values are - 0.08 watts per kilo (W/kg) averaged over the whole body - 2 W/kg locally averaged over body parts e.g. in the head **If the limit values are met, no detrimental health effects on body tissue have been established so far**. SAR values of radio waves of WLAN devices usually remain under the recommended limit value, especially when the device is far from the body. WLAN senders (2.4 GHz) in a laptop placed on a desk emitting with maximum transmitting power have local SAR-values of about 0.1 to 0.2 (W/kg). In unfavourable situations (e.g. laptop on the lap and sender immediately above the thigh), values in the dimension of the recommended limits can occur. You can find more information at www.emf-forschungsprogramm.de.	**Recommendations and precaution** 1.) Respect the minimum distances indicated by manufacturers. 2.) **The trend to portable and mobile radio applications leads to an overall increase in exposure to high-frequency electromagnetic fields. The Bundesamt für Strahlenschutz (BfS) recommends in general to minimize personal exposure in order to keep possible but not identified health risks low. Simple measures for this purpose are**: - **Prefer wired connections if wireless technology can be relinquished** - **Avoid placing central WLAN connection points in immediate proximity of places where people stay permanently, e.g. at the workplace** - **If existing, enable the distance regulation to reduce maximum radiated power**. More information regarding precautionary measures can be found by following the link www.bfs.de/elektro.

Note. Translation from German by the first author

The link http://www.bfs.de/elektro does not work anymore. It is still kept here because this is the original experimental material

The core of the precautionary principle is the obligation to base risk regulation on an ex ante approach, where precautionary actions or measures are put in place to avoid potential risks before they become definite or confirmed risks. Here, two issues are important. On the one hand, precautionary action should not be postponed until full scientific understanding of a risk issue is reached. This is especially true for uncertain risks - for which adverse effects are not proven. In other words, precautionary actions should aim to reduce potential harm from inadequately understood risks [4]. On the other hand, however, the Commission of the European Communities [5] underlines that ‘[the] precautionary principle is not a justification for ignoring scientific evidence’. According to the Commission, the principle should be invoked ‘where preliminary objective scientific evaluation, [sic!] indicates that there are reasonable grounds for concern’. In this case, precautionary actions should be proportional to the chosen level of protection [5].

With regard to implementation, the challenge is to bring RF EMF precautionary actions in line with RF EMF protection policies - usually exposure limits - that are based on scientifically identified risks. The critical issue is whether the precautionary actions might undermine trust in science-based exposure limits. Some agencies simply assume that precautionary measures align with the science-based exposure limits. For instance, Kheifets, Hester, and Banerjee [6] argue that it is possible to introduce precautionary measures without undermining trust in science-based exposure limits. However, whether that is the case is an empirical question. Previous studies (e.g. [7, 8], see below) raise some doubts. In the words of Paul Watzlawick and colleagues, precautionary actions might be part of the problem, not the solution [9].

### Effects of precautionary communication

Empirical studies have found that the communication of precautions elevates risk perceptions of its recipients [7, 8, 10–15]. While the empirical base of these findings seems robust, there are two divergent findings that need mentioning. Firstly, it has been challenged that the increase in risk perception is a specific effect of precautionary communication [16]. In that study, participants tended to have increased risk perceptions after reading EMF information brochures no matter if these brochures contained precautionary information or only other information, e.g. about technical aspects. Secondly, the effect might be more pronounced in subgroups of the population. While studies using ad-hoc and student samples mostly found an effect [7, 11, 14], a recent study only found weak indications of the effect in an Australian general population sample [17]. As a mechanism behind the effect of precautionary communication, reduction of cognitive dissonance has been discussed [8]. Stating on the one hand that the exposure limits are safe while on the other hand recommending precautions is likely to be perceived as inconsistent, a perception that can result in a state of cognitive dissonance. For a person with dissonant cognitions, a potential way of reducing the dissonance would be to dismiss the statement about the safety of the current limits and to believe that the risk is actually higher.

All of the studies capturing the effects of precautionary communication have so far used questionnaires to assess changes in risk perception and other variables (e.g. trust in public health protection) after the reception of precautionary information. These outcome variables were assessed in fictitious settings (e.g. situations without real exposure). Thus, it remains unclear to what extent a change in risk perception, i.e. the perception of RF EMFs as dangerous, expressed in a questionnaire and without being currently ‘at risk’, actually corresponds to different perceptions, cognitions, emotions or behaviour in everyday exposure situations. The current study attempts to extend existing knowledge by combining questionnaire based methods and a sham exposure paradigm. The main research question is, can precautionary communication affect participant’s symptom experiences in a situation of alleged exposure to an EMF? Whereas the practical implications of the known increase in risk perception due to precautionary information are not entirely clear [18], it would in our eyes be a clear-cut indication against the dissemination of precautionary information if a nocebo response (i.e. symptom experience under sham exposure, see next section) would be triggered by it. In this case, we would recommend authorities to reconsider their communication practice.

### Symptom experience under (sham) exposure to electromagnetic fields

An issue that remains controversial are the reports of a proportion of the population who claim to experience a range of unpleasant and debilitating non-specific symptoms when in the vicinity of devices or infrastructure which emit EMF. These individuals suffer from a condition known as Idiopathic Environmental Intolerance attributed to Electromagnetic Fields (IEI-EMF). Although it has been estimated that between 1.5 and 13.5% of the population experience this condition [19–26], the evidence to date indicates that there is no relationship between exposure to EMF and the reported symptoms [27, 28]. For instance, when tested in double-blind provocation studies, IEI-EMF participants have been shown to be unable to detect the presence of EMF and do not report an increase in symptoms to EMF [27, 28]. On the other hand, sham exposures and a person’s belief or awareness of being exposed have been found to be sufficient to trigger symptoms [28–36]. These studies underscore the importance of nocebo responses, where conscious or subconscious symptom *expectation* shapes the formation or detection of symptoms in a *perceived* EMF exposure situation.

Negative expectations about an exposure are considered to be one of the strongest predictors of a nocebo effect [37]. It is understood that these expectations may arise through explicit suggestions about the effects of an exposure [37, 38].

Interestingly, there is evidence to suggest that the manipulation of expectations via explicit suggestions about EMF exposure can induce symptoms, influence somatosensory perception and increase the likelihood of a person believing that they are sensitive to EMF in healthy participants. For example, Szemerszky, Köteles, Lihi, and Bárdos [39] demonstrated that suggestions about the strength of EMF exposure can increase symptom scores and enhance perception of a sham magnetic field. Witthöft and Rubin [40] found that viewing an inaccurate mainstream media report about potential adverse health effects of WLAN exposure increases the likelihood of a person with high pre-existing levels of state anxiety experiencing symptoms following a sham exposure and developing an apparent sensitivity to EMF. In a similar study, the researchers found that participants who watched a film focusing on ‘adverse effects of Wi-Fi’ perceived tactile electrical stimuli as more intense during a cued WLAN exposure (sham) compared to a cued no WLAN condition [41]. This effect, however, was not moderated by anxiety. To find out whether a ‘subtler’ type of information given by government agencies, namely precautionary information, can have a similar effect, is the scope of the current study.

### Hypotheses

In line with the reported effect in the study by Witthöft and Rubin [40], we propose that the effect of precautionary information on experienced symptoms will be moderated by recipient characteristics, such as personality traits or their current emotional state. That study reported an interaction effect with state anxiety, which we hypothesise as well. In addition, we also assume interaction effects with more stable recipient characteristics.

For the dependent variables ‘belief to perceive the sham EMF’, ‘difference in symptom perception’ and ‘attributed symptoms’

A. we hypothesise that the precautionary information group will have higher scores than the basic information group.

We assume this effect of information type to be more present in some recipients than others. As the interacting recipient variables we propose

B. (1) Trait anxiety, precautionary information leading to more symptom perception in highly trait anxious but not in low trait anxious individuals; (2) also, we assume that there is an equivalent interaction effect for state anxiety, as observed before [40];
C. Somatosensory Amplification (SSA), with the effect of precautionary information being present to a higher degree in individuals with higher SSA. SSA has been shown to influence nocebo responses. The construct has been conceptualised as containing three components, (a) an increased body awareness, (b) labelling minor sensations as pathological, and (c) reactions of fear or distress to these sensations [42]. It is supposed to give rise to symptom expectations and attributions [43]. The message should have no effect among those who do not tend to interpret bodily symptoms in a negative way;
D. Prior EMF risk perception, with the effect being present to a higher degree in individuals with higher prior EMF risk perception. If a person already thinks that EMFs are dangerous, she or he is more likely to interpret precautionary information as a warning sign for an existing danger.

## Methods

### Sample

Participants were recruited with two advertisements in a local newspaper, with leaflets on blackboards in supermarkets and bakeries, and by disseminating flyers at different universities in Karlsruhe as well as at a local science festival. The study was also advertised on Facebook and Twitter and on the webpage of a local TV channel. A priori power calculations with an effect size of f^2^ = .051 from a former study [40, 44] indicated that 158 participants would have to be tested for a power of 1-β = .80 in a multiple regression based analysis of the hypothesised interaction effects.

One hundred fifty seven participants took part in the study, as one participant did not show up on the penultimate day of testing. Due to noise from a nearby construction site during the first week of testing, 13 participants had to be excluded. The manipulation check of two participants revealed that they had not believed the cover story and had guessed correctly that the study was about the information material provided. They were also excluded. During testing, it turned out that four participants were not capable of fully understanding the questionnaire properly due to limited knowledge of the German language. They too were excluded. One participant withdrew from the experiment before the sham exposure.

The final sample hence consisted of 137 participants (45% females). Participant’s age distribution and their education are displayed in Table 2. It can be seen that while the aim was to recruit a sample more representative for the general population than a pure student sample, it turned out to be difficult to recruit participants aged between 30 and 50.

**Table 2:** Sociodemographic characteristics and WLAN use of the participants in the two experimental groups

	Experimental condition		Test statistic for differences between groups
	Basic information (*n* = 64)	Basic + precautionary information (*n* = 73)	
Number of females (%)	27 (42%)	35 (48%)	χ^2^ = .46 (*p* = .50)
Number of participants in age group (%)			
18–30	43 (67%)	51 (70%)	Mann-Whitney U-Test
31–40	5 (8%)	3 (4%)	Z = −.23 (*p* = 0.82)
41–50	3 (5%)	4 (6%)	
51–60	6 (9%)	7 (10%)	
older than 60	7 (11%)	8 (11%)	
Number of participants with education level (%)			
No graduation	0 (0%)	0 (0%)	Mann-Whitney U-Test
Junior high school	7 (11%)	7 (10%)	Z = −.343 (*p* = .73)
High school	26 (41%)	32 (44%)	
Bachelor degree	15 (23%)	19 (26%)	
Master degree (or equivalent)	16 (25%)	15 (21%)	
Use of WLAN at home	57 (89%)	66 (90%)	χ^2^ = .07 (*p* = .80)
Use of WLAN at work/university	49 (77%)	55 (75%)	χ^2^ = .22 (*p* = .90)

90% reported they use WLAN at home. 75% reported to use it at “work/university” (2% reported not to know whether they used WLAN at work). The achieved statistical power with 137 participants was 1-β = .75.

### Study design

The study consisted of two parts. The first part was an online survey that assessed participant variables (T_0_ questionnaire). The second part was the experiment, for which participants were randomly assigned to one of two groups using an online random number generator. During the experimental session participants read the information material and where afterwards sham exposed to an electromagnetic field of a ‘WLAN device’ in front of them, consisting of a self-constructed ‘router’ supposed to appear like a prototype and a 31.5 cm high antenna available at shops and usually used by customers as an additional antenna to strengthen reception. We experimentally varied one factor (type of information) with two factor levels (technical information including information about the safety of the current exposure limits vs. the same information plus precautionary information).

### Setting

**Fig. 1:**
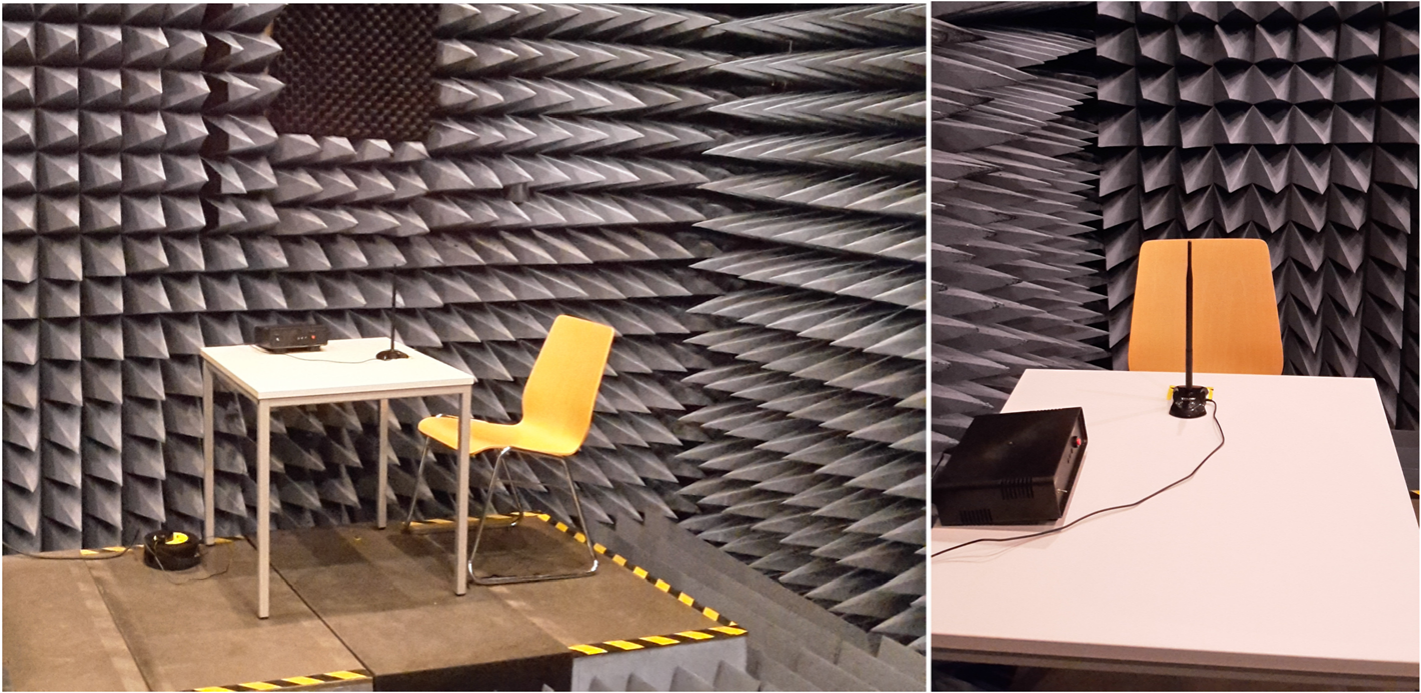
Experimental setup in the measurement room at Karlsruhe Institute of Technology

The experiment took place in a measurement room (see Fig. 1) in the basement of the university’s electrical engineering department. The measurement room is an anechoic chamber that is usually used to determine radiation characteristics of high-frequency antennas. The room is not a complete Faraday Cage, and, as described below, the door to the room was kept ajar during the experiment. The walls, floor and the ceiling are covered with pyramidal RF absorbers that absorb electromagnetic waves and also sound waves to a certain extent. Because of the latter, participants have to accommodate to the acoustics in the room (i.e. the absence of an echo).

Before running the experiment the electromagnetic power level in the room was measured to ensure that there is no relevant source of electromagnetic waves that could potentially confound the experimental design (i.e. sham EMF exposure). The power level was measured in the frequency range from 700 MHz to 6 GHz, covering the mobile radio bands, like GSM, UMTS and LTE as well as the WLAN bands around 2.45 GHz and 5.8 GHz. The measured power level was in the range of − 80 dBm (10 pW) and there was no distinct peak. This means the measured power is not a signal but a noise floor and far below the allowed 100 mW EIRP e.g. in the 2.45 GHz WLAN band.^1^

Pre-tests indicated that the room made participants think that the experiment was ‘serious’, however, they did not feel intimidated (this is also confirmed by the low state anxiety scores at T_1_ of almost all participants). A side effect of the acoustic properties of the room was that all experimenters and a large proportion of participants experienced ear noise to some extent. In the analyses, ear noise is included in the mean symptom variables reported below. However, we also conducted analyses for mean symptom variables without ear noise, but none of the results changed in terms of significance. Therefore, we only report results for the mean symptom variables including ear noise.

### Materials

#### Experimental manipulation (between T_1_ and T_2_)

The two different versions of the information about EMF are shown Table 1. The beginning of both texts contained technical information about WLAN. Both groups received the basic information but only one group received the precautionary information. The text was taken directly from an information sheet on the website of the German radiation protection agency (‘Bundesamt für Strahlenschutz’, BfS) and was modified with regard to two points only. Firstly, the original information sheet contained technical information about Blue Tooth; this information was excluded from the experimental material. Secondly, the passages about the safety of the existing limits and the precautionary information were marked in bold. The sheets containing the experimental manipulation were inserted on a clipboard in between the T1 and the T2 questionnaires by a research assistant who was otherwise not involved in the study.

#### Risk perception (T_0_ and T_3_)

As well as sociodemographic questions, the online questionnaire also comprised of four questions about EMF risk perception regarding (1) WLAN devices, (2) mobile phones while talking on the phone and (3) while transmitting data, and (4) mobile phone base stations. The items were worded ‘I consider electromagnetic fields from … dangerous for health’ and had to be answered on a five-point Likert-type answer format ranging from ‘I do not agree at all’ to ‘I fully agree’. The same questions were used again at T_3_ of the experimental part of the study.

In the online questionnaire, endurance of risk perceptions [18], i.e. the frequency of thinking about and talking about the potential health effects of EMFs was also assessed with two items each. Response scales of two questions had verbal labels ranging from ‘(almost) never’ to ‘very often’, response scales of the other two questions had numeric labels, ranging from ‘not once’ to ‘more than six times’.

#### Personality variables (T_0_)

Trait anxiety was assessed with the Trait anxiety part of the STAI Form Y [45] Somatosensory Amplification was measured with the Somatosensory Amplification Scale (SSAS, [46]). Social Desirability was assessed with the Social Desirability Scale-17 (SDS-17, [47]).

#### State anxiety (T_1_,T_2_ and T_3_)

We assessed state anxiety (SA) with the STAI-SKD [48], a 5-item version of the state part of the Spielberger state-trait-anxiety inventory.

Belief to perceive the EMF (during sham exposure), Symptoms (T_2_ and T_3_), expected symptoms (T_2_) and symptom attribution (T_3_).

The ‘belief to perceive the EMF’ was assessed after each trial of sham exposure. (1) ‘Did you perceive the electromagnetic field during this trial?’ This question had four answering options (a. ‘Yes, I am sure’; b. ‘Yes, I think so’; c. ‘No, I do not think so’; and d. ‘No, definitely not’). In the analysis, we treated this variable as a dichotome variable, with answering options a. and b. treated as ‘yes’ and options c. and d. treated as ‘no’. If participants gave answer a. or b., they also answered question (2) ‘How did you realise that there was an electromagnetic field?’ This question was answered in form of a short text or bullet points. Question 2 was not analysed in this study.

Twenty different symptoms were assessed after the experimental manipulation and before the sham exposure (T_2_) and again after sham exposure (T_3_). Participants could also list two more symptoms if they experienced something that was not on the symptom list. They rated the presence of each symptom on a 4-point Likert-type answer format ranging from ‘not at all’ to ‘strong’. Symptoms could be divided into three major groups, firstly symptoms related to head and mind (headache, dizziness, restlessness or irritability, drowsiness, fatigue, blurred vision, ear noise, dryness of the mouth, congestion of the nose, concentration difficulties), body-related symptoms (palpitation, breathlessness, breathing difficulties, muscle tension or trembling, nausea, stomach ache) and skin-related symptoms (Feeling of warmth on skin, itching of skin, prickling of skin, sweating).

Expected symptoms were assessed with the same items directly after the T_2_ symptoms, on a 5-point Likert-type answer format ranging from ‘certainly not’ to ‘certainly’. Symptom expectations were not involved in our main hypotheses. Still, as expectations are known to be a major factor in nocebo responses [37], we also assessed expectations and used it in an exploratory analysis.

To assess symptom attribution, we asked participants ‘In your opinion, to what extent were the bodily perceptions or symptoms to be ascribed to the antenna’s electromagnetic field?’ Participants answered on a 4-point Likert-type answer format ranging from ‘not at all’ to ‘to a strong extent’. There was also the additional option to choose ‘no symptoms or perceptions experienced’. If they had ascribed symptoms or perceptions to the EMF, they were supposed to list those as bullet points below.

#### Manipulation check (T_3_)

The final question of the T_3_ questionnaire was an open-ended question asking participants what they thought the experiment was about. This question acted as a manipulation check. As noted above, 2 participants were excluded because they had anticipated the study rationale. Of the 137 participants remaining after participant exclusion, 75% believed that the study was about effects of EMFs on the body or on the mind. An additional 6% thought that it was about EMF effects in conjunction with an analysis of the role of expectations or a placebo effect. The most common other answers to the manipulation check were ‘effects of prior beliefs and expectations’ (4%), a ‘placebo effect’ (3%), and answers that did not have any relation to the content (10%; e.g. ‘study for master thesis’).

### Procedure

**Fig. 2:**
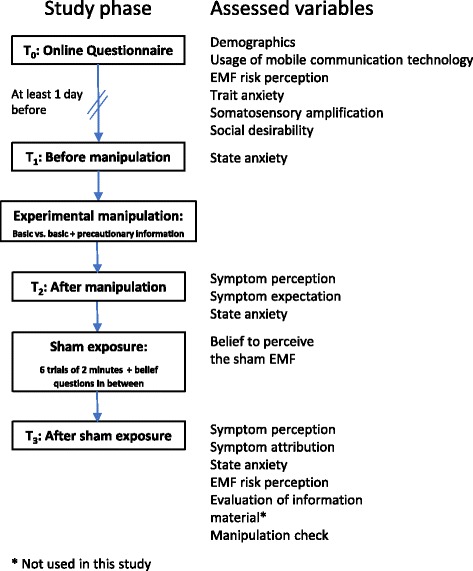
Flow of the study

Figure 2 gives a brief overview of the flow of the study and of the implemented questionnaires. Those interested in participating contacted the principal researcher or a research assistant via email or telephone. After making an appointment for the study, participants were sent an email with a link to the online questionnaire which they completed one day prior to the experiment, at the latest. Two participants received the questionnaire by mail and three participants completed the questionnaire immediately before the experiment because they had not completed it at home.

On arrival at the university, participants were first briefed about the ensuing session and signed an informed consent form. Afterwards, they were asked to turn off all electronic devices and were told about the ‘special character of the experimental room’ to which they would be led shortly, as the room ‘is shielded from outside electromagnetic fields and there are no reflections from electromagnetic fields emitted inside the room’. As the experimental room was not grounded, we provided participants with electrostatic discharge overshoes to avoid any discharge. After being asked to leave all of their belongings in an adjacent room, participants and experimenter entered the experimental room where they were seated at a table in front of the antenna and the WLAN device, which were obviously unplugged. The experimenter then explained the four stages of the experiment briefly (see below).

After this, the first stage commenced and participants were left alone in the room for two minutes ‘to accommodate to the room’. They were explicitly told to pay attention to any unusual perceptions they might have, ‘without the antenna being activated, as the room is already special’. Afterwards, the experimenter would return with a clipboard containing the T_1_ questionnaire, the information material either with or without the precautionary information (depending on randomisation), and the T_2_ questionnaires. During this second stage, participants filled out the questionnaire and read the information material. The experimenter sat down in the experimental room, approximately 2.5 m away from the participants, in order to answer any questions. In order to remain blinded to the experimental condition, experimenters pretended to read papers they had with them and avoided looking at participants while they filled out the questionnaire and read the information material. In nine cases, the experimenter did not remain blinded, most of the times because participants had a question regarding the information material. Those participants remained in the dataset, however, hypotheses A to D were additionally tested and reported without those nine cases to control for a potential bias.

After completing the questionnaire, the third stage commenced. The experimenter plugged in the WLAN device and turned it on ‘with the antenna still not being active’. The antenna was then positioned in front of the participants at a point marked with tape and the participants were asked to move the chair to a standard position as marked by tape on the floor. The participants were then asked to lean back with hands on the lap and not to touch the antenna throughout the experiment. The experimenter then explained the procedure. Participants would activate the antenna on their own, once the experimenter left the room. The door would be kept ajar throughout the experiment to ensure that communication was possible in case of any problems. When activating the antenna, the WLAN device’s green LED lights would start to flash and a short beep would sound. After two minutes, there would automatically be another beep and the LED lights would turn off, indicating that the antenna was not emitting an EMF anymore. Participants then answered two questions about their perceptions of the sham EMF (see materials section). After answering the two questions, they started the next trial by activating the antenna again. Participants were told that the antenna would emit an EMF in all trials, but that the strength of the emitted EMF would vary between the trials. If asked, the experimenter stated that exposure would always remain within the limit values set by law (this information had also already been given during the participant briefing). After the sixth and final trial, participants called the experimenter who then returned with the T_3_ questionnaire (stage four). The experimenter unplugged the antenna and removed it from its position in front of the participants and stayed in the room until participants had completed the questionnaire. After leaving the room, participants were asked if everything was alright. If they showed signs of concern about the experiment, they were debriefed immediately. If not, they were debriefed either by email (those who had not reported any symptoms) or by telephone (those who had experienced symptoms) after completion of the whole study. Finally, the experimenter handed out the monetary reimbursement and brought participants to the exit of the building. The whole experimental session lasted 45 min on average. Data were collected between May and July 2017.

### Data analysis

Main effects and the hypothesised interaction effects of personality variables and experimental group were analysed in linear multiple regressions (LMR). For that purpose, the experimental group variable was dummy-coded (with 1 referring to the precautionary information group) and the continuous independent variables were z-standardised prior to building their interaction term, as recommended by Aiken, West & Reno [49]. As the dependent variables, we used a sum score of the belief to have perceived the sham EMF, indicated right after each of the six two-minute sham exposure periods (‘belief to perceive the sham EMF’), the difference score between the mean symptom perception before and after the sham exposure (‘symptom difference T_3_-T_2_’), and a composite score that made use of the T_3_ symptom scores and participants´ attribution of symptoms to the EMF (‘attributed symptoms’). In that score, symptoms at T_3_ were only counted if participants indicated that they had attributed symptoms to some extent to the EMF. Analyses were carried out separately for each of these three dependent variables. All analyses were conducted with SPSS version 24.

In the exploratory results section, we report a mediation analysis that was conducted with Andrew Hayes´ SPSS macro PROCESS, version 2.16 [50]. Throughout the results section, we treat results with a *p*-value < .05 (two-sided test) as statistically significant. For the sake of readability, we only use the term ‘significant’, which always refers to statistical significance.

## Results

Sample characteristics are displayed in Table 2. 53 participants (39%) did not perceive the EMF in any trial while 84 participants (61%) indicated that they at least perceived the EMF in one trial. 48 participants (35%) perceived it in three or more trials. Means and standard deviations of trait anxiety, somatosensory amplification and T_0_ risk perception are shown in Table 3. The bivariate correlation between trait anxiety and somatosensory amplification was significant (r_TA, SSA_ = .27, *p* = .001). T_0_ risk perception was not correlated with the two variables (r_TA, T0RP_ = .07, *p* = .41; r_SSA, T0RP_ = .16, *p* = .06). State anxiety before the experimental manipulation was significantly higher in the precaution group than in the basic group. Because participants and experimenters were blinded, this difference can only be due to chance. This difference poses a threat to the experiments because potential group differences might not only be causally attributed to the experimental manipulation but also to the pre-existing difference in state anxiety. The difference between the two groups remained after the experimental manipulation. Symptom perceptions and their means at T_2_ and T_3_ are displayed in Appendix Table 6 in Appendix. Bivariate correlations of social desirability with independent and dependent variables were insignificant except for a correlation with T_0_ and T_3_ risk perception regarding WLAN devices (both *r* = .17, *p* < .05) and with ‘symptom difference’ (*r* = .20, *p* = .02). However, when social desirability was included as independent variable in the regressions, none of the relations between independent and dependent variables in the linear multiple regression analyses changed in terms of significance. Results are therefore reported for the equations without social desirability.

**Table 3:** Descriptive statistics of independent and dependent variables in the two experimental groups

	Experimental condition		Test statistic for differences between groups
	Basic information (*n* = 62–64)	Basic + precautionary information (*n* = 71–73)	
Independent variables	M (90% CI)	M (90% CI)	
Mean trait anxiety	2.22 (2.15–2.29)	2.23 (2.16–2.31)	t_df = 133_ = −.22 (*p* = .82)
Mean somatosensory amplification	2.71 (2.61–2.82)	2.83 (2.72–2.94)	t_df = 135_ = −1.3 (*p* = .20)
Sum social desirability	10.58 (10.05–11.10)	10.58 (10.02–11.13)	t_df = 135_ = .01 (*p* = .99)
T_0_ risk perception WLAN score	2.56 (2.33–2.79)	2.59 (2.38–2.79)	t_df = 135_ = −.14 (*p* = .89)
Mean T_1_ state anxiety	1.36 (1.29–1.42)	1.50 (1.41–1.59)	t_df = 134_ = −2.18 (p = .03)
Mean T_2_ state anxiety	1.29 (1.22–1.35)	1.42 (1.33–1.50)	t_df = 134_ = − 1.96 (*p* = .05)
Dependent variables			
Mean symptom difference T_3_ – T_2_	.09 (.04–.14)	.12 (.07–.17)	t_df = 135_ = −.65 (p = .52)
Mean attributed symptoms	1.13 (1.09–1.16)	1.15 (1.11–1.19)	t_df = 135_ = −.74 (*p* = .46)
Sum of trials with belief to perceive sham EMF format	1.53 (1.17–1.90)	2.10 (1.70–2.49)	t_df = 135_ = − 1.74 (*p* = .08)

### Effect of information and personality characteristics on symptom variables

Symptom variables were not normally distributed. However, as visual inspection of the distributions of regression residuals showed only minor deviations from the normal distribution, we did not transform symptom variables. Multicollinearity was not present in any of the regression equations (all variance inflation factors < 4). Stepwise LMR analyses showed no main effect of the experimental condition, neither on ‘symptom difference’ (b = .03, *p* = .52), nor on ‘attributed symptoms’ (b = .02, *p* = .46), nor on ‘belief to perceive the sham EMF’ (b = .57, *p* = .08). Regression weights for main effects of personality variables are reported for regressions without interaction terms. Trait anxiety was related to ‘belief to perceive the sham EMF’ (b = .34, *p* = .04, change in R^2^ = .03) with a higher trait anxiety predicting a more frequent belief. Trait anxiety was unrelated to ‘symptom difference’ (b = .03, *p* = .19) and ‘attributed symptoms’ (b = .02, *p* = .14).

State anxiety at T_2_ was related to ‘attributed symptoms’ (b = .08, *p* < .001, change in R^2^ = .19) and to ‘belief to perceive the sham EMF’ (b = .35, p = .04 change in R^2^ = .03). State anxiety at T_2_ was not related to ‘symptom difference’ (b = −.01, *p* = .65).

Somatosensory amplification was related to ‘symptom difference’ (b = .06, *p* = .007 change in R^2^ = .05) and to ‘belief to perceive the sham EMF’ (b = .65, *p* < .001 change in R^2^ = .12), with participants high in somatosensory amplification having both a higher difference in symptom perceptions and a more frequent ‘belief to perceive the sham EMF’. Somatosensory amplification was unrelated to ‘attributed symptoms’ (b = .03, *p* = .08).

T_0_ risk perception significantly predicted all three dependent variables (b = .09, *p* < .001, change in R^2^ = .13 for ‘symptom difference’; b = .04, *p* = .009, change in R^2^ = .05 for ‘attributed symptoms’ and b = .75, *p* < .001, change in R^2^ = .15 for ‘belief to perceive the sham EMF’).

There was a significant interaction between state anxiety at T_2_ and information type for ‘symptom difference’ (b = −.11, *p* = .03 change in R^2^ = .04). In the subsequent analysis of the simple slopes [49], predictions for four groups were regarded (high vs. low state anxiety; basic vs. precautionary information). Predicted symptom differences were positive for all groups, indicating that T_3_ symptom scores are predicted to be higher than T_2_ scores in all groups. Predicted symptom differences for the basic information condition were .16 for participants with high state anxiety (one standard deviation above the mean) and .04 for low state anxious participants (one standard deviation below the mean). In the precautionary information condition, predicted values were .08 for high state anxious individuals and .17 for low anxious individuals.

There were no interactions between personality variables and experimental condition (all *p* > .07).

When entering all independent variables together into one regression, explained variances rose to R^2^ = .26 for ‘symptom difference’ R^2^ = .25 for ‘attributed symptoms’, and R^2^ = .28 for ‘belief to perceive the sham EMF’. Significant predictors for ‘symptom difference’ were T_0_ risk perception (b = .10, *p* < .001) and somatosensory amplification (b = .09, *p* = .006). The only significant predictor for ‘attributed symptoms’ was state anxiety at T_2_ (b = .08, *p* = .005). The ‘belief to perceive the sham EMF’ was significantly predicted by T_0_ risk perception (b = .77, p < .001), somatosensory amplification. All other predictors and their interaction terms were insignificant.

Subsequently, the nine cases for which the experimenter did not remain blinded throughout the experiment were excluded from the data and the hypotheses were tested again. None of the results changed in terms of significance except for the interaction between somatosensory amplification and information type, which was now significant for the dependent variable ‘symptom difference’ (b = −.09, *p* = .04). Predicted symptom differences for the basic information condition were .23 for participants with high state anxiety (one standard deviation above the mean) and − .01 for low state anxious participants (one standard deviation below the mean). In the precautionary information condition, predicted values were .15 for high state anxious individuals and .10 for low anxious individuals.

To conclude, the null hypothesis was not rejected for any of the interaction effects tested.

### Exploratory analyses

Mean risk perceptions regarding WLAN at T_0_ and T_3_ are shown in Table 4. An independent samples t-Test showed that the risk perception difference between T_0_ and T_3_ did not differ between the two experimental conditions (t_df = 135_ = − 1.08, *p* = .28).

**Table 4:** Mean risk perceptions of WLAN devices before and at the end of the experiment

	Risk perception WLAN T_0_	Risk perception WLAN T_3_	Test statistic for differences between T_0_ and T_3_
	M (90% CI)	M (90% CI)	
Whole sample (N = 137)	2.58 (2.43–2.73)	2.42 (2.27–2.56)	t_df = 136_ = − 2.51 (*p* = .01)
Basic information (N = 64)	2.56 (2.33–2.79)	2.33 (2.13–2.53)	t_df = 63_ = − 2.65 (p = .01)
Precautionary information (*N* = 73)	2.59 (2.38–2.79)	2.49 (2.28–2.70)	t_df = 72_ = − 1.04 (*p* = .30)

Interestingly, mean risk perception for WLAN devices was lower at T_2_ than at T_0_ for the whole sample (*t* = − 2.51, *p* = .01). As can be seen in Table 4, this decrease was mostly driven by the basic information group.

**Fig. 3:**
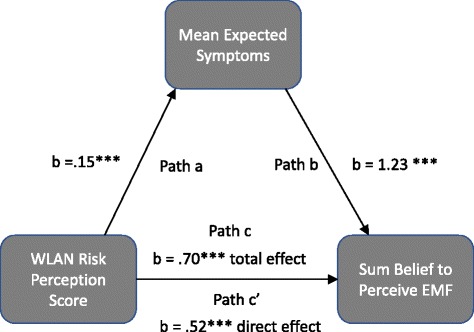
Exemplary mediation effect of T_0_ risk perception on the belief to perceive the sham EMF with symptom expectation as mediator. Note. b = bivariate regression coefficient (paths a, b and c) and semipartial regression coefficient (parth c’, with the variance of ‘mean expected symptoms’ partiallised out of ‘WLAN risk perception score’); *** = statistically significant (*p* < .001)

As T_0_ risk perception was the most powerful predictor for all three dependent variables, we analysed its effects in depth by means of a mediation analysis. The mediator in question is expected symptoms. Results from the mediation analysis can be found in Table 5. The 95% confidence intervals in Table 5 were obtained with 5000 bootstrap resamples. Figure 3 depicts the mediation. We use the nomenclature established by Baron & Kenny [51] to label the different mediation paths. Symptom expectation had a significant relationship with T_0_ risk perception (‘path a’ in the nomenclature of Baron & Kenny) as well as with all dependent variables, controlling for T_0_ risk perception (b paths). Comparisons between the total effect of T_0_ risk perception on the dependent variables (c paths) and the partial effects when controlling for symptom expectation (c’ paths) show a reduction in the size of the regression b-weights in all cases. As the c’ path b-weight remains significant for ‘symptom difference’ and ‘belief to perceive the sham EMF’, the mediation can be called a partial mediation in these cases. In the case of ‘attributed symptoms’, the c’ path b-weight does not remain significant, indicating a full mediation.

**Table 5:** Mediation analyses with T_0_ risk perception as independent variable and symptom expectation as mediator

		Coefficient	ANOVA		Sobel test	
Dependent variable	Path	b-weight; t (p)	F (p)	R^2^	Indirect effect, b-weight (95% CI)	Z (p)
Symptom difference	a	.15; 3.69 (<.001)	13.65 (< .001)	.09		
	b	.14; 3.71 (<.001)	17.65 (<.001)	.21	.02 (.008, .043)	2.57 (.01)
	c’	.06; 3.3 (.001)				
	c	.08; 4.44 (<.001)	19.70 (<.001)	.13		
Attributed symptoms	b	.16; 5.69 (<.001)	20.56 (<.001)	.23	.02 (.009, .048)	3.07 (.002)
	c’	.02; 1.09 (.28)				
	c	.04; 2.66 (.008)	7.08 (.008)	.05		
Belief to perceive sham EMF	b	1.23; 4.40 (<.001)	23.77 (<.001)	.26	.19 (.007, .364)	2.79 (.005)
	c’	.52; 3.72 (<.001)				
	c	.70; 4.98 (<.001)	24.78 (<.001)	.16

## Discussion

The present study tested whether precautionary communication regarding EMFs emitted by WLAN devices can influence symptom perceptions under sham exposure.

It was hypothesised that symptom perceptions would be higher after receiving precautionary information compared to basic technical information including a statement about the safety of the existing exposure limits. In line with existing research, it was hypothesised that the effect would be moderated by state anxiety. Additionally, it was assumed that trait anxiety, somatosensory amplification and prior risk perception would have a moderating influence. Previous studies that reported an effect of different types of information on a nocebo experience [40, 52] selected media reports that strongly suggested the harmfulness of EMFs. In contrast to these studies, the aim of the current study was to test specifically whether precautionary information, which does not directly suggest harmfulness and is disseminated by many health authorities, can also cause this effect.

Multiple regression analyses indicated that although all symptom variables were on average higher in the group that had received precautionary information, this difference was not significant. Furthermore, out of 12 tested interaction effects (with the four independent variables state anxiety, trait anxiety, somatosensory amplification and prior risk perception tested for three different dependent variables each), none of these interactions were significant or conform with the hypotheses. Thus, it can be concluded that precautionary information does not lead to increased symptom perception under a sham EMF exposure. Prior studies that found media effects on symptom perception have suggested a ‘triggering role of information in the form of written instruction or television reports’ [41], potentially leading to avoidance of EMF sources, thereby being one possible step in the development of IEI-EMF [40, 41]. Yet, whether the nocebo effect is the starting point for IEI-EMF, or whether it acts as an aggravator of pre-existing medically unexplained symptoms, as suggested previously [53], remains to be determined. As the current study did not find a short-term effect of the reception of precautionary information on symptom perception, it does probably not trigger any long-term effects by itself, either.

A special methodological feature warrants mentioning, i.e. the high ecological validity of this finding. The experimental material used in this study was original material from the German national radiation health authority (‘Bundesamt für Strahlenschutz’, BfS). Hence, it can quite reasonably be derived that the precautionary communication from the BfS does not lead to the presumably unintended effect of an increased nocebo response. Moreover, other radiation health authorities worldwide communicate in similar ways, allowing us to conclude that their communication probably does not have the hypothesised effect on its recipients, either. However, this transfer might not hold for every country that communicates precautions, (a) because the pattern of communication is often similar but never the same as the one from the BfS and (b) cultural differences might lead to a different reception process.

While there is converging evidence in the literature that precautionary information increases risk perception (see e.g. [11]), this is the second study that delineates the boundaries of this effect. In a recent study, precautionary information led to an increase in risk perception, however, the same participants did not show signs of increased state anxiety [14]. Seen from this angle, the practical relevance of EMF risk perception can be questioned. Nevertheless, prior risk perception was by far the most powerful predictor of a nocebo experience in the current study. Personality variables, namely somatosensory amplification and, to a lesser extent also trait anxiety, also predicted a nocebo experience, but had much less explanatory value. Exploratory findings show that high prior risk perception is connected to the expectation of symptoms, which in turn predict a nocebo response. This mediation, however, was only partial in two of three cases.

A sensible albeit speculative way of clarifying the role of EMF risk perception is to cast a closer look at the situation it is assessed in. In former studies, it was directly assessed after some information, either containing precautionary advice or not, had been given. Participant’s evaluation was thus directly connected to that information and the induced difference in risk perception reported in former studies might not have been sustainable. In the current study, risk perception was assessed at minimum one day before the experiment. Because of this, participants answers could be assumed to reflect the persons´ general view to a greater extent than the situational circumstances. In former studies, the effect sizes of the precautionary information on risk perception were quite small (e.g. [11, 16]). Consequently, there may also be a very small effect of precautionary information on a nocebo response. Nonetheless, statistical power in the present study was high for rather small effects, so it is very unlikely that if there was an effect, it would be of much practical relevance.

Interestingly, the average risk perception regarding EMFs from WLAN devices was lower after our experimental manipulation than before. In our eyes, this effect is probably rather due to the sham exposure situation itself than due to the information given before. As Weber [54] points out, direct experience is more likely to influence risk perceptions than any kind of information. In line with this, we think that the experience that an alleged EMF from a WLAN device does not do much harm might have outweighed any information-based effects on risk perception in our study.

Some limitations of our study need to be mentioned. Firstly, our study probably suffered from a sampling bias. People with concerns about EMFs may have been underrepresented. During recruiting, some potential participants were first interested in participating, but declined after hearing that the study was about EMFs, often muttering phrases like ‘I am already exposed enough’. It is possible that these already concerned people react stronger to precautionary information. However, we also think that among those concerned, many already know about precautions that can be taken. Therefore, the precautionary information used in this study might not have been new to them. Secondly, we chose a WLAN device as the source of the alleged EMF. The effect of precautionary information regarding other EMF sources might be different. As WLAN radiation risk perception is generally lower than mobile phone or base station risk perception [55], recipients of precautionary information regarding WLAN might not as readily react to that information as they would to precautionary information regarding other EMF sources. For instance, in the case of mobile phones, a precautionary recommendation to use a headset for mobile phone calls might – regardless of our findings – lead to a more pronounced nocebo response. In that sense, the study might suffer from a ‘floor effect’ where the supposed interaction did not manifest itself. Thirdly, and related to the second point, our exposure situation (sitting in front of a WLAN device) might not have been perceived as dangerous as the exposure situations in earlier studies that found an effect of experimental manipulation. Although 61% believed to perceive the sham EMF to some extent in our study, symptoms were generally mild. A difference due to prior reception of precautionary information might only become apparent when experiencing stronger nocebo responses.

## Conclusions

Despite these limitations, we conclude that this study can be regarded as a robust indication that precautionary information does not trigger nocebo responses. Furthermore, the absence of an interaction effect indicates that this is also true among persons who are more likely to experience a nocebo effect (i.e. people with high prior risk perception, high somatosensory amplification and high trait anxiety).

## Appendix

**Table 6:** Means of symptom perceptions at T_2_ and T_3_

Perceived symptom	T_2_ Mean (SD)	T_2_ Percent ‘markedly’; ‘strongly’^a^	T_3_ Mean (SD)	T_3_ Percent ‘markedly’; ‘strongly’ ^a^
Ear noise	2.05 (.87)	20.4; 6.6	1.98 (.95)	17.5; 8.8
Fatigue	1.35 (.58)	5.1; 0	1.57 (.76)	11.7; 1.5
Restlesness or irritability	1.28 (.51)	2.9; 0	1.25 (.58)	2.9; 1.5
Sweating	1.23 (.45)	1.5; 0	1.14 (.39)	1.5; 0
Concentration difficulties	1.22 (.53)	0.7; 1,5	1.39 (.67)	10.2; 0
Dizziness	1.21 (.43)	0.7; 0	1.39 (.68)	6.6; 1.5
Drowsiness	1.20 (.42)	0.7; 0	1.35 (.58)	5,1; 0
Palpitation	1.20 (.45)	2.2; 0	1.32 (.56)	4.4
Feeling of warmth on skin	1.18 (.44)	2.2; 0	1.26 (.61)	4.4; 1.5
Dryness of mouth	1.17 (.46)	1.5; 0.7	1.28 (.61)	5.8; 0.7
Congestion of nose	1.17 (.52)	2.2; 1.5	1.15 (.51)	2.2; 1.5
Headache	1.14 (.39)	1.5; 0	1.47 (.64)	8; 0
Blurred vision	1.11 (.34)	0.7; 0	1.21 (.56)	2.9; 1.5
Muscle tension or trembling	1.10 (.33)	0.7; 0	1.16 (.44)	2.9; 0
Breathlessness	1.10 (.35)	1.5; 0	1.17 (.49)	2.9; 0.7
Breathing difficulties	1.07 (.29)	0.7; 0	1.18 (.50)	2.9; 0.7
Prickling of skin	1.07 (.29)	0.7; 0	1.25 (.55)	5.8; 0
Nausea	1.04 (.21)	4.4; 0	1.18 (.48)	4.4; 0
Itching of skin	1.03 (.21)	0.7; 0	1.12 (.41)	2.9; 0
Stomach ache	1.01 (.09)	0.7; 0	1.14 (.42)	2.9; 0

^a^on a 4-point scale with labels 'not at ll', 'mildly', 'markedly', and 'strongly'

## Abbreviations

ANOVA: Analysis of Variance
ANSES: Agence nationale de sécurité sanitaire, de l’alimentation, de l’environnement, et du travail
ARPANSA: Australian Radiation Protection and Nuclear Safety Authority
BAG: Bundesamt für Gesundheit
BfS: Bundesamt für Strahlenschutz
CI: Confidence Interval
EIRP: Equivalent Isotropic Radiated Power
EMF(s): Electromagnetic Field(s)
GHz: Gigahertz
GSM: Global System for Mobile Communications
IARC: International Agency for Research on Cancer
ICNIRP: International Commission on Non-Ionizing Radiation Protection
IEI-EMF: Idiopathic Environmental Intolerance attributed to Electromagnetic Fields
LAN: Local Area Network
LED: Light-Emitting Diode
LMR: Linear Multiple Regressions
LTE: Long Term Evolution
MHz: Megahertz
RF-EMFs: Radiofrequency Electromagnetic Field(s)
SA: State Anxiety
SAR: Specific Absorption Rate
SDS-17: Social Desirability Scale-17
SPSS: Statistical Package for Social Sciences
SSA: Somatosensory Amplification
SSAS: Somatosensory Amplification Scale
STAI: State Trait Anxiety Index
TV: Television
UMTS: Universal Mobile Telecommunications System
WHO: World Health Organization
WLAN: Wireless Local Area Networks

## Footnotes

1 In response to the request of a reviewer the power level in the frequency range from 1 MHz to 700 MHz was measured. For these measurements, the measured power level was also in the range of − 80 dBm (10 pW) and there was no distinct peak.
